# Up-Regulation of Nerve Growth Factor in Cholestatic Livers and Its Hepatoprotective Role against Oxidative Stress

**DOI:** 10.1371/journal.pone.0112113

**Published:** 2014-11-14

**Authors:** Ming-Shian Tsai, Yu-Chun Lin, Cheuk-Kwan Sun, Shih-Che Huang, Po-Huang Lee, Ying-Hsien Kao

**Affiliations:** 1 Department of Surgery, E-DA Hospital, Kaohsiung, Taiwan; 2 The School of Medicine for Post-Baccalaureate, I-Shou University, Kaohsiung, Taiwan; 3 Department of Medical Research, E-DA Hospital, Kaohsiung, Taiwan; 4 Department of Medical Education, E-DA Hospital, Kaohsiung, Taiwan; 5 Department of Surgery, National Taiwan University Hospital, National Taiwan University College of Medicine, Taipei, Taiwan; CIMA. University of Navarra, Spain

## Abstract

The role of nerve growth factor (NGF) in liver injury induced by bile duct ligation (BDL) remains elusive. This study aimed to investigate the relationship between inflammation and hepatic NGF expression, to explore the possible upstream molecules up-regulating NGF, and to determine whether NGF could protect hepatocytes from oxidative liver injury. Biochemical and molecular detection showed that NGF was up-regulated in cholestatic livers and plasma, and well correlated with systemic and hepatic inflammation. Conversely, systemic immunosuppression reduced serum NGF levels and resulted in higher mortality in BDL-treated mice. Immunohistochemistry showed that the up-regulated NGF was mainly localized in parenchymal hepatocytes. In vitro mechanistic study further demonstrated that TGF-β1 up-regulated NGF expression in clone-9 and primary rat hepatocytes. Exogenous NGF supplementation and endogenous NGF overexpression effectively protected hepatocytes against TGF-β1- and oxidative stress-induced cell death in vitro, along with reduced formation of oxidative adducted proteins modified by 4-HNE and 8-OHdG. TUNEL staining confirmed the involvement of anti-apoptosis in the NGF-exhibited hepatoprotection. Moreover, NGF potently induced Akt phosphorylation and increased Bcl-2 to Bax ratios, whereas these molecular alterations by NGF were only seen in the H_2_O_2_-, but not TGF-β1-treated hepatocytes. In conclusion, NGF exhibits anti-oxidative and hepatoprotective effects and is suggested to be therapeutically applicable in treating cholestatic liver diseases.

## Introduction

Cholestatic liver injury is not an uncommon clinical scenario, which can be caused by obstructed bile flow due to sclerosing cholangitis, periampullary tumor, cholelithiasis and prolonged parenteral nutrition use [1]. The pathological changes of liver associated with cholestasis include hepatocyte necrosis and apoptosis, neutrophil infiltration, bile duct epithelial proliferation, hepatic stellate cell activation and finally fibrosis. Production of reactive oxygen species (ROS) is among the key factors underlying liver injury [2], [3].

Nerve growth factor (NGF) is vital for the differentiation, survival, and synaptic activity of the peripheral sympathetic and sensory nervous systems [4], [5]. Moreover, NGF is up-regulated in various types of inflamed tissues [6] and shown to protect nerve cells against oxidative stress [7], [8], [9], [10]. In our previous study, gastric perforation enhanced aortic as well as cardiac expression of both NGF mRNA and protein [11]. In liver, NGF has been demonstrated to play a role in regulating liver fibrosis [12], [13], [14], carcinogenesis [15], [16], angiogenesis [15], and cholangiocyte proliferation [17]. In response to various chemical injuries, NGF expression is up-regulated in the liver [18]. Although NGF has been reported to be up-regulated during experimental cholestatic injury [17], its role in hepatocytes following oxidative injury and its mechanism of regulation during cholestasis remain unclear. Moreover, little is known about the underlying mechanisms mediating NGF effects on hepatocytes.

In the present study, we hypothesized that cholestatic injury can up-regulate NGF expression in liver through an inflammatory signaling axis. We further investigated whether NGF is able to exert anti-apoptotic effects on hepatocytes and protect hepatocytes from various insults, including oxidative stress. We showed that NGF induced activation of PI3K/Akt and up-regulated the Bcl-2/Bax ratios in hepatocytes. Furthermore, NGF protects hepatocytes against TGF-β1 and hydrogen peroxide-induced oxidative damage. These data shed new light on the mechanism whereby NGF provides protection against oxidative injury and may be potentially relevant in the development of new therapeutic modalities for cholestatic liver injury.

## Materials and Methods

### Animals and ethics statement

Six to eight-week-old ICR male mice were raised *ad libitum* at 20–22°C with a 12 hr of light-dark cycle in the Animal Center of I-Shou University. All animal experimental procedures were approved by the Institute of Animal Care and Use Committee at E-DA Hospital (Affidavit of Approval of Animal Use Protocol No. IACUC-99018 and 100015) and performed in accordance with the Guide for the Care and Use of Laboratory Animals (NIH publication No. 85–23, National Academy Press, Washington, DC, USA, revised 1996). Mice were randomly divided into experimental groups. Cholestatic liver injury was induced by surgical procedures for common bile duct ligation (BDL) as previously described [19]. In brief, induction of anesthesia of mice was performed by inhalation of a gas mixture of 2.5% isoflurane and oxygen. After laparotomy under deep anesthesia, the common bile duct was doubly ligated and transected between the two ligatures and followed by abdominal closure with absorbable sutures. Postoperative analgesia was immediately performed by single subcutaneous injection with Ketoprofen at 5 mg/kg. For time-course observation, six mice were used for each time point. Specimens were collected at day zero for normal control and on 7 and 14 post operative days (POD) for BDL groups. For anti-inflammatory treatment, methylprednisolone sodium succinate (MP; Solu-Medrol, Pharmacia & Upjohn Company, New York, NY) or normal saline as solvent control was intraperitoneally administered under anesthesia at a dose of 5 mg/kg daily immediately after BDL surgery for 14 consecutive days. Six mice in each experimental group were used to observe survival and specimens were collected from survivors at end point.

### Serum and liver tissue collection

At the time points indicated, 1.5 mL of whole blood was collected from the mice under deep anesthesia with inhalation of isoflurane, followed by direct percutaneous puncture of left ventricle. After centrifugation, sera were frozen at −80°C until analysis. Serum samples were used to determine biochemical parameter levels, including aspartate aminotransferase (AST), alanine aminotransferase (ALT) and total bilirubin, through a clinical automatic analyzer (Department of Laboratory Medicine, E-DA Hospital). Liver tissues were dissected and aliquoted into three parts for mRNA, protein and paraffin-embedded tissue sectioning.

### ELISA

Serum cytokine levels were determined using commercially available ELISA kits (TNF-α and IL-6 from Biolegend, San Diego, CA; NGF from Millipore, Billerica, MA; TGF-β1 from R&D, Minneapolis, MN) according to manufacturer's instructions.

### Reverse transcription (RT) and quantitative polymerase chain reaction (qPCR)

Total RNA was extracted from liver tissues or cultured cells using Trizol reagent (Invitrogen, Gaithersburg, MD). Two micrograms of total RNA was subject to RT-qPCR analysis as previously described [19]. In brief, an AMV reverse transcriptase system (Promega, Madison, WI) was used to generate complementary DNA. Real-time PCR amplification was performed on a thermal cycler (ABI 7500, Applied Biosystems, Foster City, CA) using the FastStart DNA Master^PLUS^ SYBR Green I kit (Roche, Castle Hill, Australia) under the following cycling conditions: one cycle of 95°C for 10 min, 45 cycles of 95°C for 15 s, 60°C for 5 s, and 72°C for 20 s. An extra melting curve protocol was used at the final step to validate specificity of PCR reaction. The primer sequences were: *β-actin*, 5′-TCC TGT GGC ATC CAC GAA ACT-3′ (forward) and 5′-GAA GCA TTT GCG GTG GAC GAT-3′ (reverse); *NGF*, 5′- ACG CAG CTT TCT ATC CT-3′ (forward) and 5′- TTT AGT CCA GTG GGC TTC-3′ (reverse); *TGF-β1*, 5′-CGT CAG ACA TTC GGG AAG C-3′ (forward) and 3′-CAG CCA CTC AGG CGT ATC A-3′ (reverse); *TNF-α*, 5′- TGA ACT TCG GGG TGA TCG GTC -3′ (forward) and 5′- AGC CTT GTC CCT TGA AGA GAA -3′ (reverse); *IL-6*, 5′- ATG AAC AAC GAT GAT GCA CTT G -3′ (forward) and 5′- TAA GTC AGA TAC CTG ACA ACA G -3′ (reverse). Parallel amplification of *β-actin* was used as the internal control. Fold change of each gene was calculated by the comparative Ct method.

### Western blotting analysis

Liver tissues and cellular total proteins were extracted with an ice-cold RIPA lysis buffer containing protease inhibitor cocktail (Roche, Indianapolis, IN) and phosphatase inhibitors (1 mM sodium fluoride and 1 mM sodium orthovanadate), followed by protein measurement using a Coomassie protein assay kit (Pierce Biotechnology, Rockford, IL). SDS-PAGE, electrotransfer, and immunodetection were performed as previously described [20]. For detection, antibodies against NGF, 4-hydroxynonenal (4-HNE) and 8-hydroxydeoxyguanosine (8-OHdG) were from Millipore (Temecula, CA), TGF-β1, Bcl-2, Bax, phosphor- and total Akt were from Cell Signaling (Danvers, MA), and β-actin from Santa Cruz (Santa Cruz, CA); Bcl-2 and Bax from Trevigen (Gaithersburg, MD). For the purpose of semi-quantitative analysis, images of enhanced chemiluminescent signal were digitally documented on an imaging system (BioSpectrum, UVP, Upland, CA) and densitometrically analyzed using ImageJ software (NIH, USA). Relative protein levels were expressed as induction folds by calculating the density ratios between interest proteins and internal control and normalizing to negative control. For determination of cellular levels of oxidative adducts, densities of the major immunoreactive signals ranging from 40 to 90 kDa and those from 50 to 70 kDa were summed up and considered as formation of 4-HNE and 8-OHdG-related protein adducts, respectively. Fold changes were calculated by normalization with respective internal controls and expressed as folds of negative control.

### Immunohistochemistry (IHC)

Formalin-fixed and paraffin-embedded mouse liver sections were used for IHC staining as previously described [20]. Briefly, the deparaffinized and rehydrated sections were treated for antigen retrieval and incubated with anti-NGF polyclonal antibodies (1∶200 dilution) at 4°C overnight. The antigenicity in tissue sections was visualized with an HRP-linked polymer Envision detection system (DAKO, Glostrup, Denmark) followed by hematoxylin counterstaining. Normal liver sections treated with normal rabbit IgG at equimolar concentration were used as negative controls.

### Cell culture and viability assay

For cytokine stimulation experiments, primary hepatocytes were isolated from male Fisher 344 rats (220-260 g) using a two-step collagenase perfusion method as previously described [21]. Primary hepatocytes were plated onto the plates pre-coated with type I collagen at a density of 5×10^5^ cells/well. For NGF gene transfection, clone-9 hepatocytes (BCRC no. 60201) were purchased from Bioresource Collection and Research Center (Hsin-Chu, Taiwan) and maintained in F-12K medium (Sigma) with 10% heat-inactivated fetal calf serum (Invitrogen, Logan, UT) and regular antibiotics [19]. For cell viability determination, an MTT-based cellular assay was performed as previously described [22].

### NGF gene cloning, plasmid construction, and gene transfection

Full length NGF cDNA was cloned from a human fetal brain cDNA library (Stratagene, La Jolla, CA) by PCR reaction. The PCR primers used to clone the human full-length NGF cDNA (1052 bp) were designed based on the NGF sequence in the GenBank database (accession number, NM_002506; using 5′- CCG CTC GAG AGA GAG CGC TGG GAG C -3′ as forward primer, and 5′- TCC CCC GGG TTT ATG CTT CCA AAA -3′ as reverse primer). The PCR-amplified NGF cDNA was cloned into the pCR-Blunt II-TOPO vector and transformed into E. coli competent cells provided in the Zero Blunt TOPO PCR cloning kit (Invitrogen, Carlsbad, CA). After DNA sequencing analysis, the full length NGF cDNA fragment was subcloned into the *Xho*I and *Sma*I sites of pCMS-EGFP mammalian expression vector with enhanced green fluorescent protein (pCMS-EGFP, Invitrogen), thus giving rise to a recombinant plasmid containing NGF cDNA (pCMS-NGF). Afterwards, the transformed cells were grown at 37°C until log phase (OD_600 nm_∼0.5–0.9). The plasmid DNA was amplified and prepared for in vitro gene transfection using a liposome-based gene delivery system (Lipofectamine 2000, Invitrogen) according to manufacturer's instructions. Briefly, 1∼4 µg plasmid DNA and Lipofectamine solution were mixed under serum-reduced condition and added into cultured cells at 70% confluency. After 24 hrs of incubation, the medium was replaced with fresh medium containing G418. The overexpressed GFP was observed under fluorescent microscopy, whereas the NGF gene transfection efficiency was assessed at both transcriptional and translational levels by using RT-qPCR and ELISA assays, respectively.

### Apoptotic detection by TUNEL staining

Primary rat hepatocytes grown on chamber slides were fixed with ice-cold paraformaldehyde after receiving treatment, and subjected to a TUNEL-based in situ cell death detection assay. TUNEL signals were detected and visualized with DAB color formation using a standard protocol provided by manufacturer (Roche, Mannheim, Germany). Slides were counterstained with hematoxylin. Quantification of the nuclear positive signals in each group was performed by counting at least 20 randomly selected images at high-power fields under microscopy and the positivity was shown in percentage of total cells.

### Statistics

In vivo data were presented as mean±standard error of mean (SEM), while in vitro as mean±standard deviation (SD). Significance among groups was determined by one-way analysis of variance (ANOVA) followed by the Bonferroni post hoc test. A *p* value less than 0.05 was considered statistically significant.

## Results

### Up-regulated NGF expression in parenchymal hepatocytes of cholestatic livers

**Figure 1:**
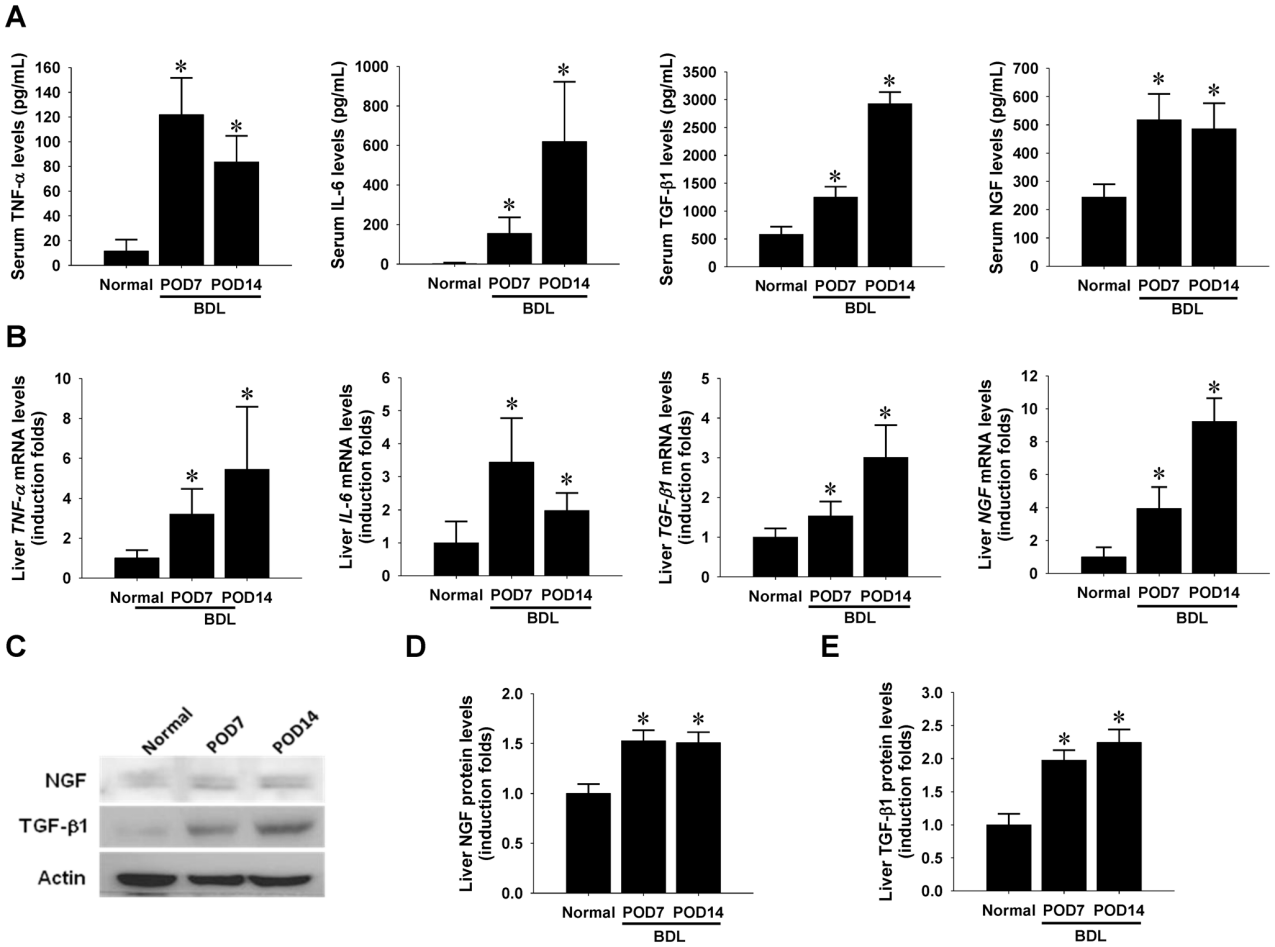
Increased serum NGF levels and intrahepatic NGF up-regulation in mice receiving bile duct ligation (BDL) surgery. Normal mice (n = 6) and those receiving BDL surgery were sacrificed at 7 or 14 post-operative days (POD7, n = 4) or (POD14, n = 4). (A) Serum levels of NGF and inflammatory cytokines, including TNF-α, IL-6 and TGF-β1 were determined by ELISA detection. (B) Liver tissue extracts were collected for mRNA isolation and subjected to RT-qPCR analysis. (C) Contents of NGF and TGF-β1 proteins in pooled liver extracts in experimental groups were measured using Western blot detection. Subsequent densitometrical analysis showed that cholestatic injury increased protein abundance of NGF (D) and TGF-β1 (E). All data are shown in mean±SEM. * indicates *P* <0.05 as compared to normal controls.

To elucidate the role played by NGF in cholestatic liver injuries, mice serum and liver tissues were collected at 7 days and 14 days post BDL operation. Elevated plasma levels of AST, ALT, and total bilirubin confirmed the effectiveness of surgery-induced cholestatic injury in mouse livers (**Figure S1**). ELISA detection showed that the pro-inflammatory cytokine levels including TNF-α, IL-6, and TGF-β1 in those mice with liver injury were significantly elevated. In parallel, the plasma NGF levels also increased along with the progression of liver fibrosis (**Figure 1A**). To investigate whether the cholestatic insult triggers de novo synthesis of NGF in livers, total RNA and protein extracts were used for further molecular measurements. RT-qPCR analysis indicated that the intrahepatic transcript contents of *TNF-α*, *IL-6, TGF-β1,* and *NGF* genes were remarkably up-regulated at POD14 of BDL surgery (**Figure 1B**), while gene expression patterns correlated with those of serum peptides. Western blotting demonstrated a similar increasing trend between intrahepatic NGF and TGF-β1 peptides (**Figure 1C, 1D, 1E**).

### Localization of up-regulated NGF expression in liver parenchymal hepatocytes

**Figure 2:**
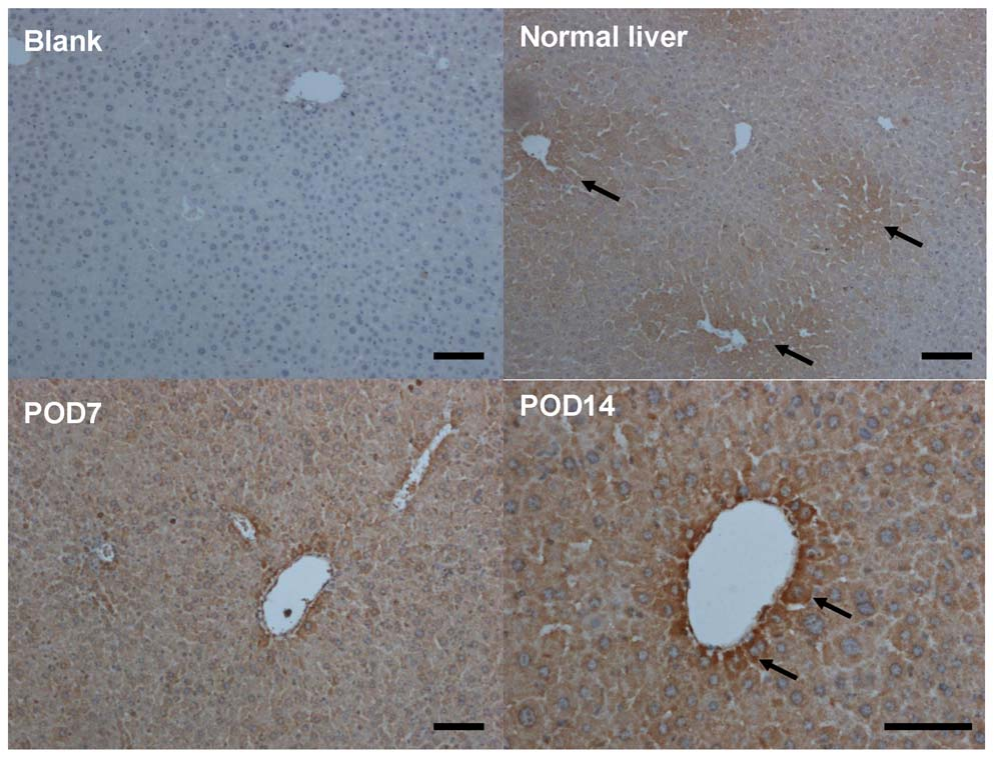
Parenchymal localization of NGF peptide in hepatocytes with cholestatic injury. The mice receiving bile duct ligation surgery were sacrificed at POD7 and POD14 and the liver tissues were fixed with formalin. Paraffin-embedded tissue sections were subjected to immunohistochemisty NGF staining. Arrows indicate the parenchymal localization of NGF antigenicity in zone 3. Bars, 50 µm.

To better characterize NGF localization in normal and injured livers, formalin-fixed and paraffin-embedded liver sections were applied to histopathological examinations. IHC staining results for NGF peptides showed that, in normal liver, NGF was expressed at constitutively lower levels and its antigenic signal was not homogeneously distributed in all liver lobules but only seen in limited areas around the central vein zone 3 (**Figure 2**). After cholestatic injury, the constitutive NGF antigenicity level was apparently up-regulated and homogeneously distributed throughout the hepatic lobules at POD7. Again, stronger NGF antigenicity was seen mainly localized to the cytoplasms of parenchymal hepatocytes in injured livers at POD14. The above findings highlight the significance of the NGF up-regulation in liver fibrosis and suggest that it may play a pathophysiological role therein.

### Amelioration of systemic and intrahepatic inflammation and suppression of NGF up-regulation by MP treatment

**Figure 3:**
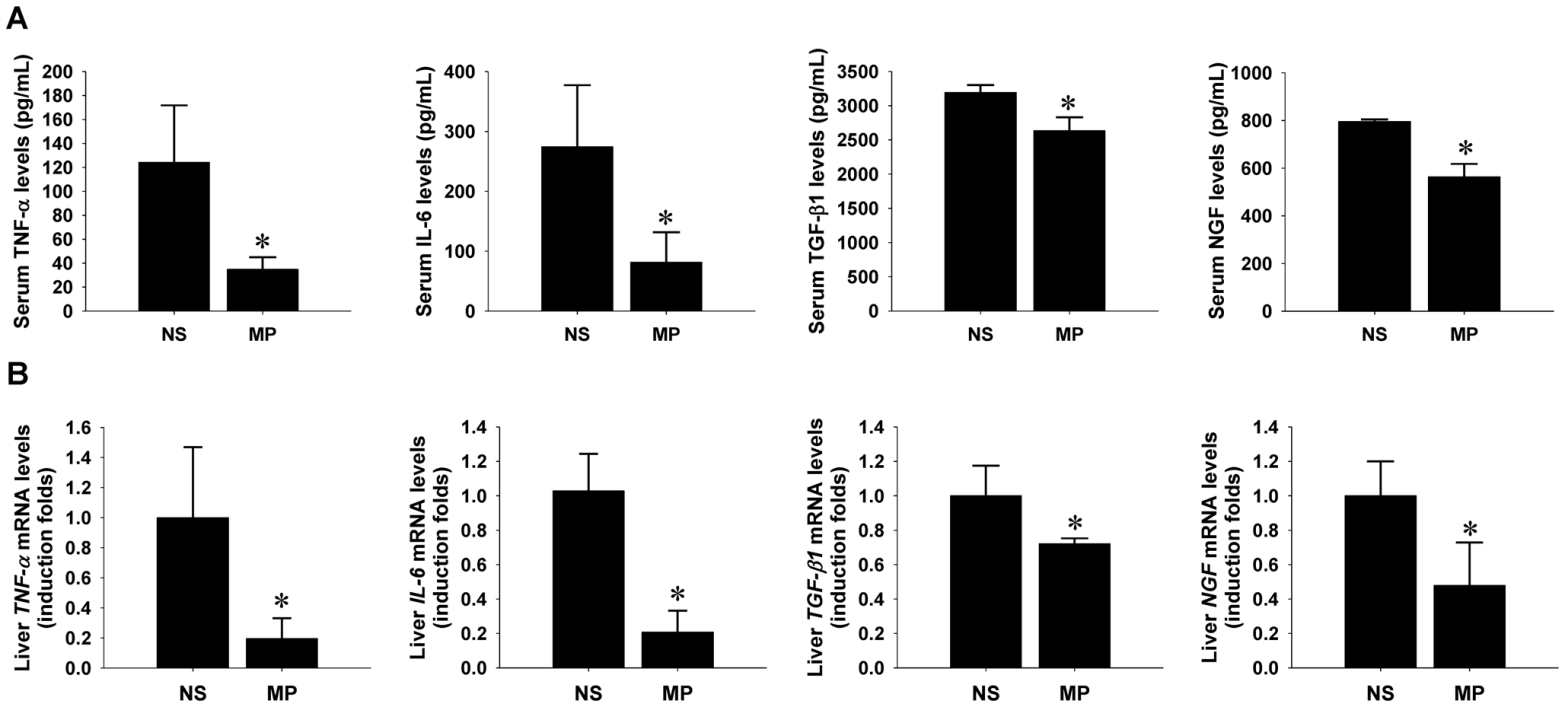
Amelioration of systemic inflammation and NGF up-regulation in cholestatic liver-bearing mice by methylprednisolone (MP). The mice receiving bile duct ligation surgery immediately underwent intraperitoneal administration with either normal saline (NS, n = 5) or MP (n = 3) at 1 mg/kg/day for 14 consecutive days. Mice plasma and liver extracts were collected and subjected to ELISA (A) and RT-qPCR analyses (B), respectively. Inflammatory cytokines measured included TNF-α, IL-6, NGF, and TGF-β1. Data are shown in mean±SEM. * indicates *P*<0.05 compared to NS group.

Since NGF has been previously reported to be up-regulated by inflammatory cytokines in diseased bladder [6], aorta [23], heart [11], and livers [17], [18], [24], [25], we next to determine whether anti-inflammatory treatment could ameliorate the cholestasis-associated NGF up-regulation in injured livers. A synthetic glucocorticoid drug (MP) was used to suppress systemic immunactivity and thereby clarify the causal relationship between inflammation and systemic and/or hepatic NGF up-regulation. Biochemistry data showed that MP treatment effectively suppressed AST (**Figure S2A**), but not the elevated plasma levels of ALT (**Figure S2B**) and total bilirubin (**Figure S2C**). Although Western blotting revealed that the MP treatment did not affect NGF and TGF-β1 peptide contents in cholestatic livers (**Figure S2D**), ELISA data demonstrated that MP prominently reduced serum levels of TNF-α, IL-6, TGF-β1, and NGF peptides in the surviving mice with cholestatic liver injury (**Figure 3A**). Similarly, RT-qPCR data also showed that MP treatment remarkably lowered the transcript contents of *TNF-α*, *IL-6*, *TGF-β1*, and *NGF* genes in injured livers (**Figure 3B**). These findings strongly suggested that MP administration not only systemically suppressed host immunity but also locally lowered NGF de novo synthesis in livers, which might, at least in part, underlie the reduction of NGF contents in plasma pools. More intriguingly, systemic immunosuppression by MP administration immediately after BDL surgery caused a higher mortality (3 out of 6) compared to that in normal saline controls (1 out of 6) (**Figure S3**), implicating that NGF may play a hepatoprotective role in livers and that suppression of systemic NGF levels at an acute stage may aggravate cholestatic injury and be lethal.

### Up-regulation of NGF in rodent hepatocytes by TGF-β1

**Figure 4:**
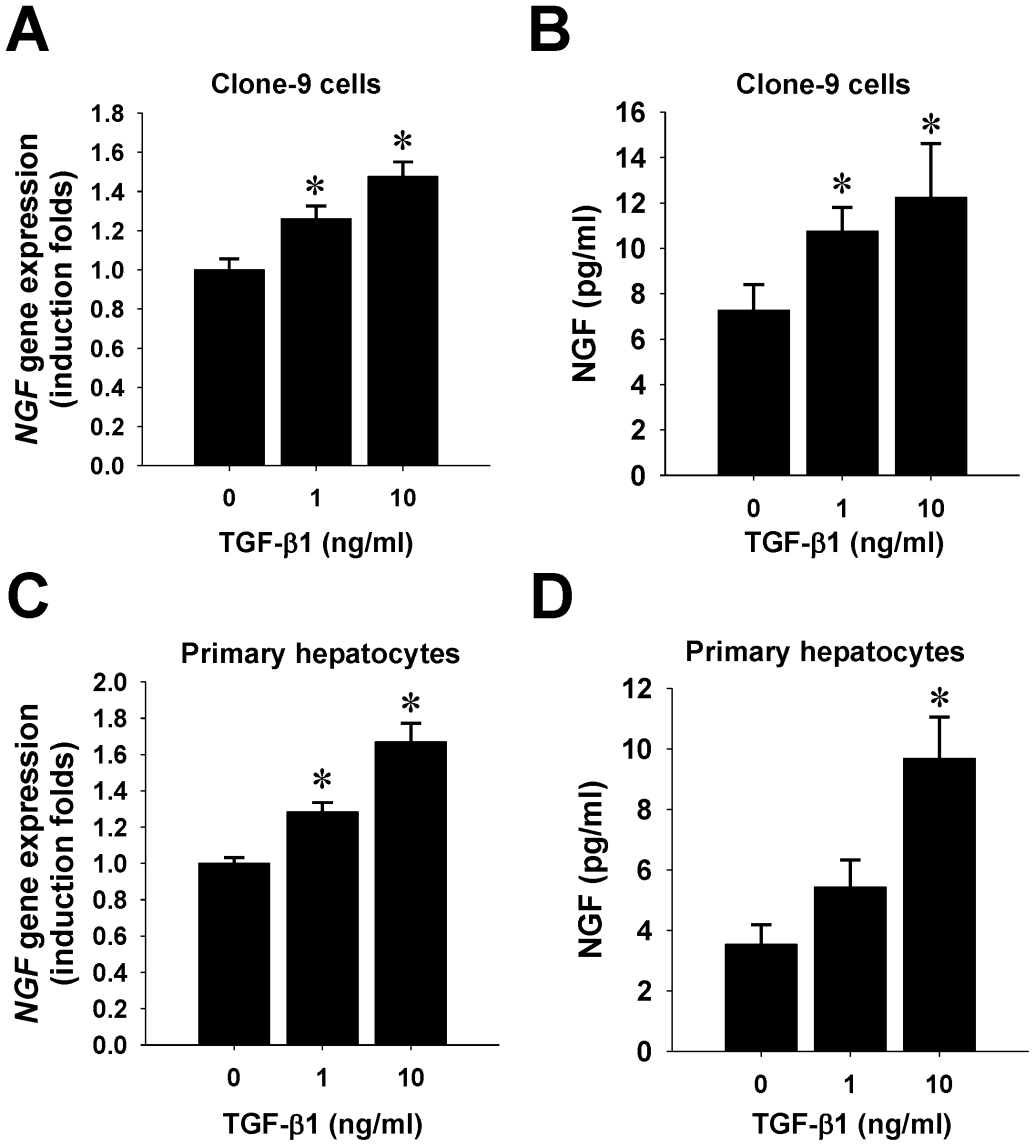
Up-regulation of NGF expression in rodent hepatocytes by TGF-β1. Both clone-9 and primary rat hepatocytes were used for NGF induction experiments. Clone-9 cells (A, B) and primary hepatocytes isolated from rats (C, D) were grown on collagen I-coated dishes and treated with TGF-β1 at the indicated doses (ng/mL) for 6 h. Total RNA was extracted and subjected to qPCR analysis for *NGF* mRNA levels (A, C). Besides, conditioned media for 24 hrs of treatment were collected for NGF ELISA detection (B, D). Note that TGF-β1 at 10 ng/mL remarkably increased de novo synthesis of NGF in both cultured hepatocytes. Data are representative results from three independent experiments and shown in mean±SD. * indicates *P*<0.05 compared to negative control group.

Since pro-inflammatory cytokines such as TGF-β1 have been demonstrated to up-regulate NGF expression in pancreatic stellate cells [26], we next sought to answer whether TGF-β1 or other pro-inflammatory cytokines are responsible for the NGF up-regulation in parenchymal hepatocytes of cholestatic livers. The result of a pilot cytokine screening showed that TNF-α, IL-1β, and IL-6 did not stimulate NGF gene transcription in primary rat hepatocytes (**Figure S4**). To further determine the regulatory role of TGF-β1, a line of clone-9 hepatocytes and primary rat hepatocytes were treated with TGF-β1 and the NGF expression was quantified at both transcription and translation levels. The RT-qPCR data revealed that exogenous TGF-β1 significantly increased NGF gene transcripts in both clone-9 cells (**Figure 4A**) and primary hepatocytes (**Figure 4C**). Similarly, ELISA for conditioned media also showed that exogenous TGF-β1 significantly increased soluble NGF peptide release from both clone-9 cells (**Figure 4B**) and primary hepatocytes (**Figure 4D**), supporting that NGF expression in parenchymal hepatocytes was up-regulated by TGF-β1.

### Exogenous NGF treatment attenuated TGF-β1- and H_2_O_2_-induced hepatotoxicity and oxidative stress

**Figure 5:**
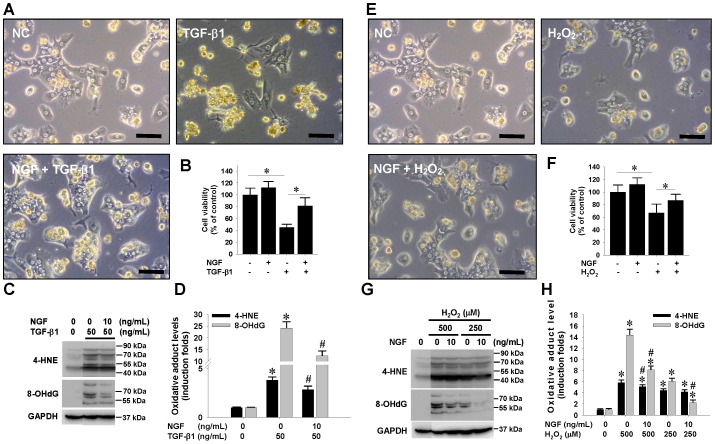
In vitro hepatoprotective effects of exogenous NGF supplementation on TGF-β1-induced and oxidative cell death. Primary rat hepatocytes were treated with recombinant NGF at 20 ng/mL for 24 hrs and exposed to TGF-β1 and H_2_O_2_ for another 24 hrs, followed by morphological observation (A, E) and cell viability assay (B, F). Representative microphotographs were shown (*Bar*  = 50 µm). Cellular viability was determined by the MTT-based viability assay. Western blots (C, G) and subsequent densitometrical analyses (D, H) show that NGF pretreatment attenuated the elevation of 4-HNE and 8-OHdG modified protein levels induced by TGF-β1 and H_2_O_2_. Data are representative results from three independent experiments and expressed as mean±SD. * indicates *P*<0.05, as compared between groups or with negative control. # indicates *P*<0.05 compared with corresponding NGF-negative groups.

To better understand the hepatoprotective effect of NGF, primary rat hepatocytes were pretreated with recombinant NGF for 24 hrs, followed by treatment with either TGF-β1 or H_2_O_2_. Morphological observation (**Figure 5A, 5E**) and simultaneous cell viability evaluation demonstrated that pretreatment with exogenous NGF significantly rescued both the TGF-β1- (**Figure 5B**) and H_2_O_2_-elicited hepatotoxicity (**Figure 5F**). Since NGF was previously reported to play an anti-oxidative role in the nervous system [10], [27], [28], we next determined whether NGF protects hepatocytes through ameliorating oxidative stresses, NGF-pretreated hepatocytes were exposed to TGF-β1 and H_2_O_2_ insults and the intracellular levels of the proteins injured by 4-HNE and 8-OHdG adducts were determined. Western blotting and subsequent densitometrical results clearly showed that NGF pretreatment prominently suppressed the elevation of 4-HNE and 8-OHdG modified protein levels in hepatocytes induced by TGF-β1 (**Figure 5C, 5D**) and H_2_O_2_ (**Figure 5G, 5H**). Parallel Western detection confirmed the existence of two NGF receptors, TrkA and p75 NTR, in primary hepatocytes (**Figure S5**), supporting the integrity of NGF signaling machinery wherein.

### Endogenous NGF overexpression protected cultured hepatocytes against TGF-β1- and H_2_O_2_-induced cell death and oxidative stresses

**Figure 6:**
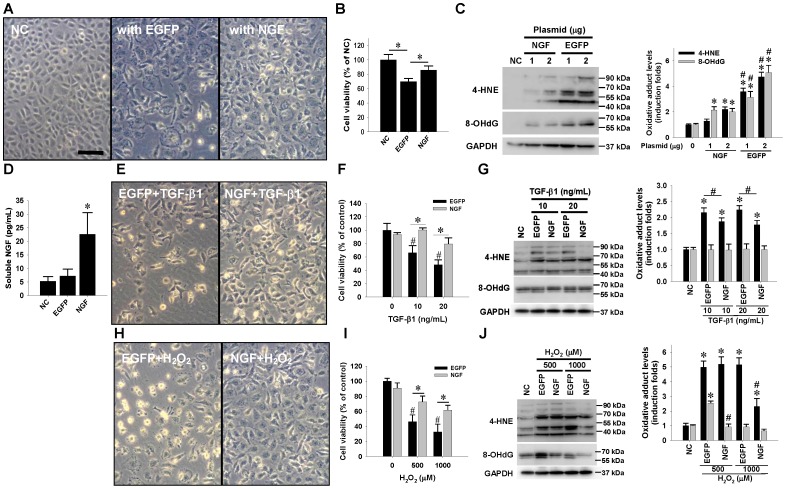
In vitro hepatoprotective effects of endogenous NGF overexpression on TGF-β1-induced and oxidative cell death. Clone-9 hepatocytes were transfected with either pCMS plasmid encoding EGFP (EGFP) or full-length NGF cDNA (NGF) using Lipofectamine reagent for 48 hrs, followed by morphological documentation (A, E, H) and viability determination (B, F, I). *Bar*  = 50 µm. ELISA showed that NGF gene transfection for 48 hrs significantly induced soluble NGF production in conditioned medium (D). The clone-9 hepatocytes transfected with plasmids were exposed to either TGF-β1 or H_2_O_2_ for 24 hrs. The MTT cell viability assay showed that NGF overexpression not only prevented transfection- induced cytotoxicity (B) but also reduced TGF-β1 (F) and H_2_O_2_ (I) cytotoxicity. # and * indicate *P*<0.05 compared with negative control (NC) and between groups, respectively. Western blotting results showed that NGF overexpression attenuated the elevation of cellular oxidative adduct formation, including 4-HNE and 8-OHdG modified proteins, induced by plasmid transfection (C), TGF-β1 (G), and H_2_O_2_ insults (J). Data are representative results from three independent experiments, and normalized to NC. Density data are expressed as mean±SD. * indicates *P*<0.05 compared with NC; # indicates *P*<0.05 compared with corresponding EGFP- or NGF-transfected group.

To mimic NGF overexpression in hepatocytes, clone-9 hepatocytes were transfected with pCMS plasmids carrying either EGFP or full-length NGF cDNA. ELISA confirmed that NGF gene delivery after 48 hrs of transfection drove clone-9 cells to significantly produce soluble NGF peptides (**Figure 6D**). We next tested the ability of NGF to affect viability in response to hepatotoxic insults. Transfection with EGFP plasmid alone reduced hepatocyte viability, while NGF overexpression prevented transfection-induced cytotoxicity as revealed by cell morphological observation (**Figure 6A**) and a cell viability assay (**Figure 6B**). Not surprisingly, transfection with plasmids expressing EGFP gave rise to higher cellular levels of both 4-HNE and 8-OHdG adduct-modified proteins than those in the cells overexpressing NGF (**Figure 6C**). To determine whether NGF overexpression ameliorated the pro-apoptotic and oxidative stimuli, NGF-overexpressing clone-9 cells were further treated with either TGF-β1 or H_2_O_2_ treatment for 24 hrs. Morphological observation clearly showed that NGF overexpression reduced the TGF-β1- and H_2_O_2_-induced cytotoxicity (**Figure 6E, 6H**). The cell viability assay consistently confirmed the NGF-driven hepatoprotective effects against both insults (**Figure 6F, 6I**). Again, NGF overexpression effectively reduced the increased formation of both oxidative adducts caused by TGF-β1 and H_2_O_2_ treatment (**Figure 6G, 6J**).

### Anti-apoptosis was involved in NGF-mediated hepatoprotection against oxidative stress

**Figure 7:**
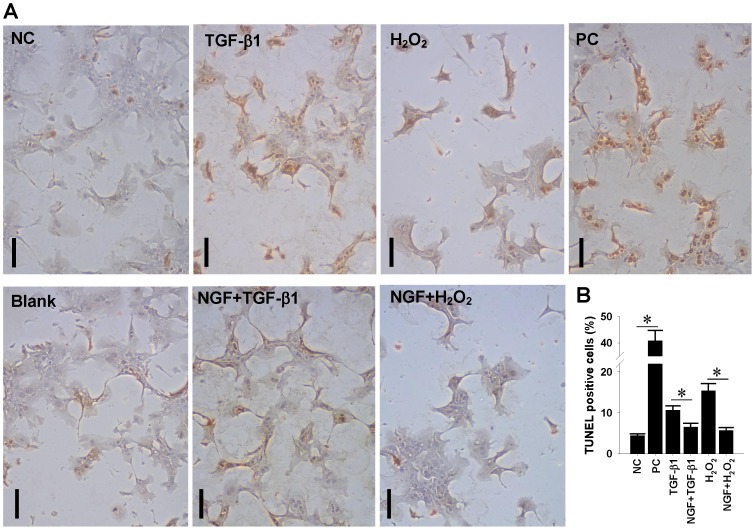
Anti-apoptotic effect of exogenous NGF pretreatment on TGF-β1-induced and oxidative cell death of primary hepatocytes. Primary rat hepatocytes grown on collagen-coated chamber slides were treated with recombinant NGF at 20 ng/mL for 24 hrs and exposed to either TGF-β1 at 50 ng/mL or H_2_O_2_ at 500 µM for another 24 hrs. After treatments, cells were fixed with paraformaldehyde and subjected to in situ TUNEL staining for cellular apoptotic events. (A) Representative TUNEL staining images are shown (Bars  = 10 µm). Positive control (PC) was performed by treating cells with DNase I. (B) Nuclear TUNEL-positive signals were quantified by measuring 20 high-power fields per group and the positivity are shown in mean±SEM. * indicates *P*<0.05 as compared with negative control (NC) or between groups.

To confirm the involvement of anti-apoptogenesis in the NGF-exhibited hepatoprotection against oxidative stress, in situ TUNEL detection was used to quantify the cellular apoptotic events under in vitro hepatotoxic injury. The TUNEL staining (**Figure 7A**) and quantitative results (**Figure 7B**) clearly indicated that both TGF-β1 and H_2_O_2_ insults significantly increased nuclear apoptotic signals in treated primary rat hepatocytes, while NGF pretreatment effectively prevented the increased hepatocytic apoptosis induced by both agents, supporting that NGF may functionally protect hepatocytes against TGF-β1- and oxidation-induced hepatocellular apoptosis.

### Involvement of NGF-induced Akt phosphorylation and increase of Bcl-2/Bax ratios in hepatocytes under oxidative stress

**Figure 8:**
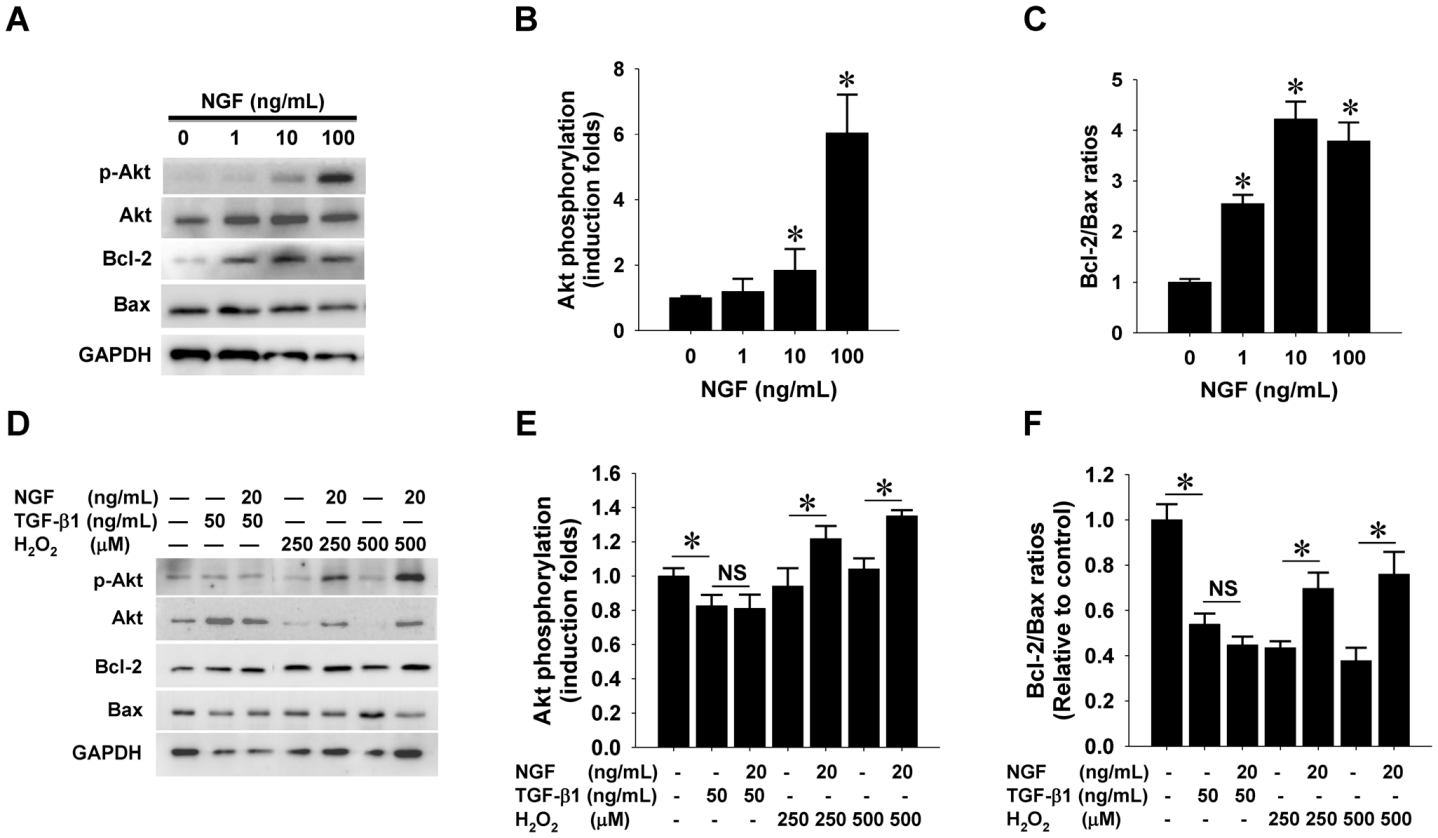
In vitro biomodulatory effect of NGF in cultured hepatocytes. (A) Clone-9 hepatocytes were treated with recombinant NGF for 24 hrs at indicated doses. Protein lysates were collected and subjected to Western blot detection for phosphor-Akt, total Akt, Bcl-2 and Bax expression levels. The relative Akt phosphorylation (B) and the ratios of Bcl-2 to Bax protein levels (C) were densitometrically measured. (D) Primary rat hepatocytes were exposed to TGF-β1 with or without 24 hrs of NGF pretreatment and the lysates were subjected to Western blotting. Subsequent densitometry showed that NGF pretreatment significantly increased Akt phosphorylation in the cells with H_2_O_2_, but not TGF-β1 insult (E). Similarly, NGF prominently ameliorated the down-regulated ratio of Bcl-2 and Bax proteins only in the cells with H_2_O_2_ exposure (F). Data are representative results from three independent experiments and shown as mean±SD. * indicates *P*<0.05 compared with negative control or between groups. NS, not significant.

Since the NGF-up-regulated Bcl-2 expression is responsible for its anti-oxidative ability in the nervous system [29], we next examined whether NGF modulates anti- and/or pro-apoptotic machineries in hepatocytes in vitro. Western blotting results showed that NGF at a dose of 10 ng/mL or higher for 24 hrs significantly induced phosphorylation of Akt (**Figure 8A, 8B**), the upstream mediator of Bcl-2. Concomitantly, NGF enhanced expression of anti-apoptotic protein Bcl-2, but suppressed that of pro-apoptotic protein Bax. Taken together, the Bcl-2-to-Bax ratios were significantly increased (**Figure 8C**). To further determine whether NGF supplementation ameliorates the disruption of anti-apoptotic machinery induced by TGF-β1 and H_2_O_2_, primary hepatocytes with or without NGF pretreatment were under exposure to either insult. Western blotting indicated that the NGF-up-regulated Akt phosphorylation (**Figure 8D, 8E**) and the increased Bcl-2-to-Bax ratios (**Figure 8D, 8F**) were only seen in the cells with H_2_O_2,_ oxidative insult, but not in those with TGF-β1 treatment. These findings support that the up-regulated NGF in injured livers possesses anti-apoptotic benefit for hepatocyte survival through activating Akt signaling and restoring the equilibrium between Bcl-2 and Bax.

## Discussion

Using a cell line and primary culture hepatocytes, this study is the first to show the hepatoprotective ability of NGF against oxidative stress and TGF-β1, both being key mediators in cholestatic liver injury [1], [19]. Moreover, we also demonstrated that cholestasis-related inflammatory signaling such as TGF-β1 was able to induce NGF expression in cultured hepatocytes. The findings in the present study improved our understanding about the pathobiological role of NGF and the molecular mechanisms in the pathogenesis of cholestatic liver injury.

### Enhanced hepatic expression of NGF during cholestasis

Local or systemic inflammation has been shown to induce NGF production in various tissue types, including the bladder [6], aorta [23] and heart [11]. NGF up-regulation in livers has been previously demonstrated under a wide range of pathological scenarios, including hepatotoxin-induced fibrosis [24], ischemia-reperfusion injury [25], oxidative injury [18], and cholestatic injury [17]. Consistent to the findings of previous studies, the present study demonstrated that BDL resulted in enhanced hepatic expression of both NGF mRNA and protein, which is temporarily related to the elevation of inflammatory cytokines in both liver extracts and plasma, including IL-6, TNF-α, and TGF-β1 (**Figure 1**). This observation implicates that inflammatory signaling may serve as an upstream player to modulate hepatic NGF expression. Although systemic anti-inflammation by MP did not effectively reduce hepatic content of NGF peptides (**Figure S2**), we still noted that it prominently suppressed the up-regulated NGF transcription in livers and the elevated NGF peptides in plasma of the BDL animals (**Figure 3**). Together with the fact that soluble NGF could be released from the NGF-overexpressing hepatocytes (**Figure 6D**), all the evidence supports the concept that hepatic NGF production is induced by inflammation signaling and eventually contributes to the systemic pool in this rodent BDL model. Moreover, it is worth to emphasize that the systemic immunosuppression by MP treatment immediately after BDL surgery resulted in not only a lower plasma NGF levels but also a higher mortality (**Figure S3**). This result may, at least in part, reflect the hepatoprotective effect of systemic NGF peptides and highlight again the biological significance of the NGF up-regulation during the acute stage of liver injury.

Our in vitro mechanistic study further demonstrated that TGF-β1 but not IL-6 or TNF-α, stimulated NGF production by hepatocytes in a dose-dependent manner (**Figure 4**). Consistently, other lines of evidence also showed that TGF-β1 up-regulates NGF in pancreatic stellate cells [26] and dental pulp cells[30], while the signaling pathways involved include activin-like kinase-5 [26] and mitogen-activated protein kinase [30]. Moreover, we also observed that NGF could induce the expression of TGF-β1 in hepatocytes (data not shown). In fact, a mutual regulation between TGF-β1 and NGF has been previously noted in the nervous system [31], [32]. Further studies are warranted to explore the biological significance and the mechanisms underlying the mutual regulation of TGF-β1 and NGF in parenchymal hepatocytes.

### Anti-oxidative and hepatoprotective effects of NGF on hepatocytes

**Figure 9:**
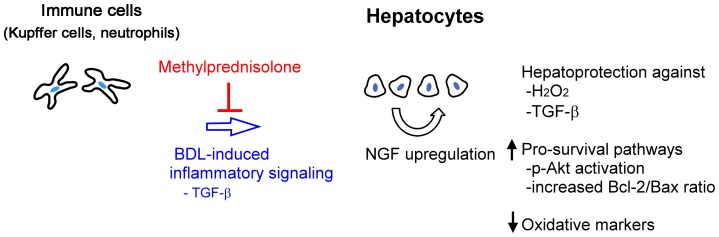
Hypothetical scheme showing the regulatory mechanisms and hepatoprotective roles of NGF. When the liver encounters BDL-induced cholestatic injury, inflammatory signals will induce NGF up-regulation, which can be blocked by systemic MP administration. In vitro study shows that TGF-β1 may be one of the upstream molecules that induce NGF expression in parenchymal hepatocytes. NGF is able to (1) ameliorate hepatocyte cell death caused by exogenous hydrogen peroxide and TGF-β1, (2) enhance pro-survival pathways, including p-Akt and Bcl-2/Bax ratio, (3) decrease intracellular oxidative adduct formation.

Oxidative stress plays an important role in the cellular interactions, and is crucial during the pathogenesis of cholestatic liver injury and fibrosis [33], [34]. ROS production can result from activation of resident macrophages (Kupffer cells) and recruitment of neutrophils and monocytes into the liver [2], [35]. Moreover, cytokines released from inflammatory cells and accumulation of bile acids per se can increase oxidative stress and/or suppress anti-oxidative machinery within hepatocytes [36], [37], [38]. Upon exposure to oxidative stress, hepatocytes may develop several mechanisms, including impaired mitochondrial function, activation of the Akt pathway and disequilibrium of Bcl-2/Bax ratio [3]. Despite these intrinsic defense mechanisms, persistent accumulation of ROS within the liver will inevitably cause hepatocyte cell death and eventually promote fibrosis [38]. In this study, we demonstrated that exogenous NGF supplement and endogenous overexpression protected cultured rodent hepatocytes against hydrogen peroxide-induced hepatocellular death (**Figure 5**). Meanwhile, the oxidative markers, including 4-HNE and 8-OHdG-modified protein levels, were significantly reduced in the NGF-overexpressing cells (**Figure 6**). The hepatocellular protection of exogenous NGF supplement was demonstrated to be mediated through its anti-apoptotic effect (**Figure 7**). Mechanistically, the NGF-enhanced Akt phosphorylation and recovered Bcl-2/Bax ratios were involved therein (**Figure 8**). We thus propose that NGF is able to activate various cellular mechanisms of not only anti-oxidative stress but also cell survival (**Figure 9**). As for the contradictory result that NGF supplement did not change status of Akt phosphorylation and Bcl-2/Bax ratios (**Figure 8D**) but effectively ameliorated TGF-β1-induced cytotoxicity (**Figure 5**), it is most likely that different sets of apoptotic regulators may participate in different apoptogenic scenarios. In fact, not only Bcl but also inhibitor of apoptosis protein (IAP) family members could confer resistance to the induced hepatocyte apoptosis [36], [39], [40]. This issue awaits further elucidation.

In the context of NGF-elicited anti-oxidative effect, NGF deprivation has long been known to induce oxidative stress in the nervous system [27]. Moreover, NGF also inhibited the oxidative stress-induced apoptosis of PC12 cells [10], [28]. The anti-oxidative effect of NGF is related to increased expression of Bcl-2 protein family members [29], activation of mammalian target of rapamycin (mTOR) signaling [7], phospho-Akt pathway [41] and up-regulation of free radical scavenging enzymes [28]. In line with our findings, oxidative stress itself was more recently reported to up-regulate NGF mRNA expression in livers, while functional blockade of NGF with a neutralizing antibody increased hepatic oxidative stress and decreased glutathione production [18], supporting an intimately regulatory relationship between NGF and oxidative stress. Moreover, exogenous NGF treatment was shown to increase hepatocyte intracellular glutathione levels through the TrkA signaling pathway [42]. It will be interesting to further elucidate the exact mechanisms involved in the hepatoprotective effects of NGF.

All the different liver component cells under cholestatic insult must adjust their cell behaviors and modulate interactions with one another accordingly. In addition to the anti-oxidative and hepatoprotective effects, NGF also plays multifunctional roles in an autocrine or paracrine manner and modulates fibrogenesis and liver regeneration during liver injuries. For instance, NGF, also reportedly produced by cholangiocytes, was demonstrated to promote cholangiocyte proliferation [17], which is one of the hallmarks of cholestasis. Another well-studied effect of NGF is to induce apoptosis of hepatic stellate cells and consequently resolve liver fibrosis [14], [43], [44]. These studies, along with our findings, highlight the pathobiological significance of NGF among different types of cells within normal and diseased livers. Although the present study demonstrated the molecular mechanisms of NGF regulation and its protective effects in hepatocytes, more studies are needed to determine whether the pathological changes of cholestasis could be ameliorated through manipulation of NGF receptor signal axis in vivo.

### Study limitations

A few limitations of this study should be addressed. First, although we showed TGF-β1, but not IL-6 or TNF-α, enhanced NGF expression in vitro, it does not mean that TGF-β1 is the only upstream molecule regulating NGF or it is sufficient alone to regulate NGF in vivo. Experimental animal studies using TGF-β1 knock-out or knock-in animals may be helpful to clarify the causal relationship. Second, the downstream effects of NGF depend greatly on the type of its receptors. TrkA signaling tends to be cytoprotective, while p75 usually mediates proapoptotic effects. It is generally believed that hepatocytes mainly express TrkA receptors [41], [42], whereas p75 is a marker for hepatic stellate cells [12], [43]. Although we confirmed the existence of TrkA and p75 receptors in both normal liver parenchyma (**Figure S6**) and cultured primary hepatocytes (**Figure S5**), to date it is still unclear whether and how the expression of NGF receptors changes during liver injury. Therefore, to elucidate which receptor is responsible for the NGF-exhibited hepatoprotection is imperative to further understand the role of NGF in various liver diseases.

In conclusion, the present study demonstrates that NGF up-regulation is related to the inflammatory process during experimental cholestatic liver injury. We also found, for the first time, NGF is able to protect hepatocytes against oxidation-induced hepatocellular death, along with amelioration of cellular oxidative stress. Therefore, NGF supplementation is suggested to be therapeutically applicable in cholestatic liver injury.

## Supporting Information

Figure S1 **Serum biochemistry data from mice after cholestatic injury.** Normal mice (n = 6) and those receiving BDL surgery were sacrificed at 7 or 14 post-operative days (POD7, n = 4) or (POD14, n = 4). Collected mouse sera were subjected to biochemical measurements, including AST (A), ALT (B), and total bilirubin (C). Gray boxes represent quartile deviation of groups. Data are shown in mean±SEM. * indicates *P*<0.05 as compared to the normal control.
(DOC)

Figure S2 **Intrahepatic NGF protein expression was not changed by methylprednisolone (MP) treatment.** The mice receiving bile duct ligation surgery underwent intraperitoneal administration with either normal saline (NS, n = 5) or MP (n = 3) at 5 mg/kg/day for 14 days. Mice sera were collected and subjected to biochemical analyses, including AST (A), ALT (B), and total bilirubin (C). The liver tissue were collected for protein isolation and subsequently subjected to Western blotting detection (D). Note that MP treatment only suppressed serum AST levels. Although MP treatment significantly reduced plasma NGF levels, it did not prevent the cholestasis-induced NGF up-regulation in livers. Data are shown in mean±SEM. * indicates *P*<0.05 compared to NS group.
(DOC)

Figure S3 **Effect of methylprednisolone (MP) treatment on survival of mice with experimental cholestatic liver injury.** Normal saline (NS) was used as solvent control group.
(DOC)

Figure S4 **TNF-α, IL-1β and IL-6 did not induce NGF expression.** Primary hepatocytes were isolated from rats and grown on collagen I-coated dishes. Cells were treated with TNF-α, IL-1β, and IL-6 at the indicated doses (ng/mL) for 6 hrs. Total RNA was extracted and subjected to qPCR analysis for *NGF* mRNA levels. Expression levels were normalized to internal control actin gene. Note that none of cytokines increased transcription of NGF gene in cultured primary hepatocytes. Data are shown in mean±SD. * indicates *P*<0.05 compared to the negative controls (NC).
(DOC)

Figure S5 **Expression levels of TrkA and p75NTR in primary rat hepatocytes.** Primary hepatocytes isolated from rat livers were treated with either recombinant TGF-β1 at 50 ng/mL or H_2_O_2_ at 500 µM for 24 hrs. Protein lysates were collected and subjected to Western blot detection for TrkA and p75NTR expression.
(DOC)

Figure S6 **Immunohistochemistry showing parenchymal localization of TrkA and p75NTR peptides in normal mouse livers.** Formalin-fixed and paraffin-embedded mouse liver tissues were subjected to immunohistochemical staining and counterstaining with hematoxylin. Note that homogenous pattern and sparsely spotted distribution of TrkA and p75 NTR, two NGF cognate receptors, were seen in parenchyma of normal mouse livers. Images at right panel are the magnified rectangular area indicated by dashed lines in left images. Bars  =  100 µm.
(DOC)
