# Supplementary material for: Up-Regulation of Nerve Growth Factor in Cholestatic Livers and Its Hepatoprotective Role against Oxidative Stress
Source: PLoS One. 2014 Nov 14;9(11):e112113. doi: 10.1371/journal.pone.0112113 (PMC4232375; doi:10.1371/journal.pone.0112113)
Supplement: Figure S1 — Serum biochemistry data from mice after cholestatic injury. Normal mice (n = 6) and those receiving BDL surgery were sacrificed at 7 or 14 post-operative days (POD7, n = 4) or (POD14, n = 4). Collected mouse sera were subjected to biochemical measurements, including AST (A), ALT (B), and total bilirubin (C). Gray boxes represent quartile deviation of groups. Data are shown in mean±SEM. * indicates P<0.05 as compared to the normal control. (DOC) [file pone.0112113.s001.doc]

**A B C**

**Figure S1** Serum biochemistry data from mice after cholestatic injury. Normal mice (n=6) and those receiving BDL surgery were sacrificed at 7 or 14 post-operative days (POD7, n=4) or (POD14, n=4). Collected mouse sera were subjected to biochemical measurements, including AST (A), ALT (B), and total bilirubin (C). Gray boxes represent quartile deviation of groups. Data are shown in mean±SEM. * indicates *P* < 0.05 as compared to the normal control.
