# Supplementary material for: Up-Regulation of Nerve Growth Factor in Cholestatic Livers and Its Hepatoprotective Role against Oxidative Stress
Source: PLoS One. 2014 Nov 14;9(11):e112113. doi: 10.1371/journal.pone.0112113 (PMC4232375; doi:10.1371/journal.pone.0112113)
Supplement: Figure S2 — Intrahepatic NGF protein expression was not changed by methylprednisolone (MP) treatment. The mice receiving bile duct ligation surgery underwent intraperitoneal administration with either normal saline (NS, n = 5) or MP (n = 3) at 5 mg/kg/day for 14 days. Mice sera were collected and subjected to biochemical analyses, including AST (A), ALT (B), and total bilirubin (C). The liver tissue were collected for protein isolation and subsequently subjected to Western blotting detection (D). Note that MP treatment only suppressed serum AST levels. Although MP treatment significantly reduced plasma NGF levels, it did not prevent the cholestasis-induced NGF up-regulation in livers. Data are shown in mean±SEM. * indicates P<0.05 compared to NS group. (DOC) [file pone.0112113.s002.doc]

**A B**

**C D**


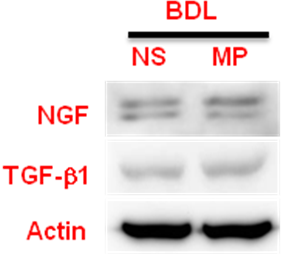


**Figure S2.** Intrahepatic NGF protein expression was not changed by methylprednisolone (MP) treatment. The mice receiving bile duct ligation surgery underwent intraperitoneal administration with either normal saline (NS, n=5) or MP (n=3) at 5 mg/kg/day for 14 days. Mice sera were collected and subjected to biochemical analyses, including AST (A), ALT (B), and total bilirubin (C). The liver tissue were collected for protein isolation and subsequently subjected to Western blotting detection (D). Note that MP treatment only suppressed serum AST levels. Although MP treatment significantly reduced plasma NGF levels, it did not prevent the cholestasis-induced NGF up-regulation in livers. Data are shown in mean±SEM. * indicates *P*<0.05 compared to NS group.
