# Supplementary material for: Up-Regulation of Nerve Growth Factor in Cholestatic Livers and Its Hepatoprotective Role against Oxidative Stress
Source: PLoS One. 2014 Nov 14;9(11):e112113. doi: 10.1371/journal.pone.0112113 (PMC4232375; doi:10.1371/journal.pone.0112113)
Supplement: Figure S3 — Effect of methylprednisolone (MP) treatment on survival of mice with experimental cholestatic liver injury. Normal saline (NS) was used as solvent control group. (DOC) [file pone.0112113.s003.doc]

**Figure S3** Effect of methylprednisolone (MP) treatment on survival of mice with experimental cholestatic liver injury. Normal saline (NS) was used as solvent control group.
