# Supplementary material for: Up-Regulation of Nerve Growth Factor in Cholestatic Livers and Its Hepatoprotective Role against Oxidative Stress
Source: PLoS One. 2014 Nov 14;9(11):e112113. doi: 10.1371/journal.pone.0112113 (PMC4232375; doi:10.1371/journal.pone.0112113)
Supplement: Figure S4 — TNF-α, IL-1β and IL-6 did not induce NGF expression. Primary hepatocytes were isolated from rats and grown on collagen I-coated dishes. Cells were treated with TNF-α, IL-1β, and IL-6 at the indicated doses (ng/mL) for 6 hrs. Total RNA was extracted and subjected to qPCR analysis for NGF mRNA levels. Expression levels were normalized to internal control actin gene. Note that none of cytokines increased transcription of NGF gene in cultured primary hepatocytes. Data are shown in mean±SD. * indicates P<0.05 compared to the negative controls (NC). (DOC) [file pone.0112113.s004.doc]

**Figure S4.** TNF-α, IL-1β and IL-6 did not induce NGF expression. Primary hepatocytes were isolated from rats and grown on collagen I-coated dishes. Cells were treated with TNF-α, IL-1β, and IL-6 at the indicated doses (ng/mL) for 6 hrs. Total RNA was extracted and subjected to qPCR analysis for *NGF* mRNA levels. Expression levels were normalized to internal control actin gene. Note that none of cytokines increased transcription of NGF gene in cultured primary hepatocytes. Data are shown in mean±SD. * indicates *P*<0.05 compared to the negative controls (NC).
