# Supplementary material for: Up-Regulation of Nerve Growth Factor in Cholestatic Livers and Its Hepatoprotective Role against Oxidative Stress
Source: PLoS One. 2014 Nov 14;9(11):e112113. doi: 10.1371/journal.pone.0112113 (PMC4232375; doi:10.1371/journal.pone.0112113)
Supplement: Figure S5 — Expression levels of TrkA and p75NTR in primary rat hepatocytes. Primary hepatocytes isolated from rat livers were treated with either recombinant TGF-β1 at 50 ng/mL or H2O2 at 500 µM for 24 hrs. Protein lysates were collected and subjected to Western blot detection for TrkA and p75NTR expression. (DOC) [file pone.0112113.s005.doc]

**Figure S5.** Expression levels of TrkA and p75NTR in primary rat hepatocytes. Primary hepatocytes isolated from rat livers were treated with either recombinant TGF-β1 at 50 ng/mL or H_2_O_2_ at 500 μM for 24 hrs. Protein lysates were collected and subjected to Western blot detection for TrkA and p75NTR expression.
