# Supplementary material for: Up-Regulation of Nerve Growth Factor in Cholestatic Livers and Its Hepatoprotective Role against Oxidative Stress
Source: PLoS One. 2014 Nov 14;9(11):e112113. doi: 10.1371/journal.pone.0112113 (PMC4232375; doi:10.1371/journal.pone.0112113)
Supplement: Figure S6 — Immunohistochemistry showing parenchymal localization of TrkA and p75NTR peptides in normal mouse livers. Formalin-fixed and paraffin-embedded mouse liver tissues were subjected to immunohistochemical staining and counterstaining with hematoxylin. Note that homogenous pattern and sparsely spotted distribution of TrkA and p75 NTR, two NGF cognate receptors, were seen in parenchyma of normal mouse livers. Images at right panel are the magnified rectangular area indicated by dashed lines in left images. Bars = 100 µm. (DOC) [file pone.0112113.s006.doc]

**TrkA**


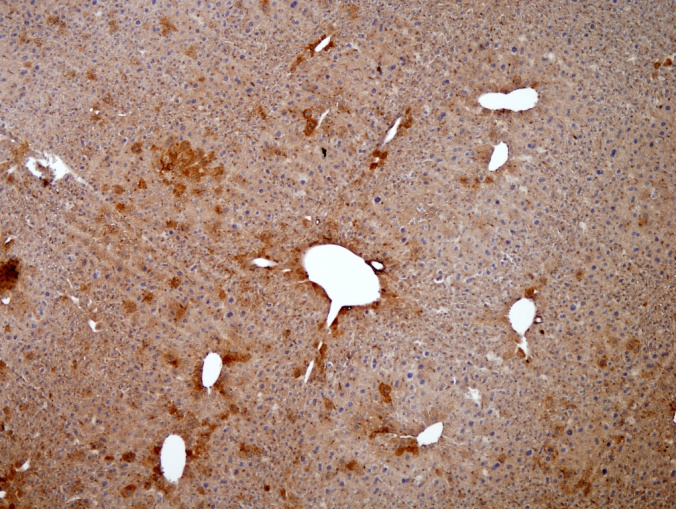

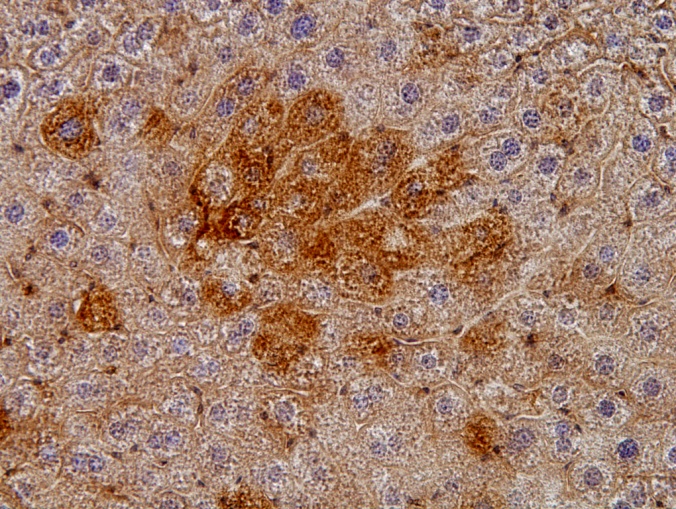


**TrkA**

**p75NTR**


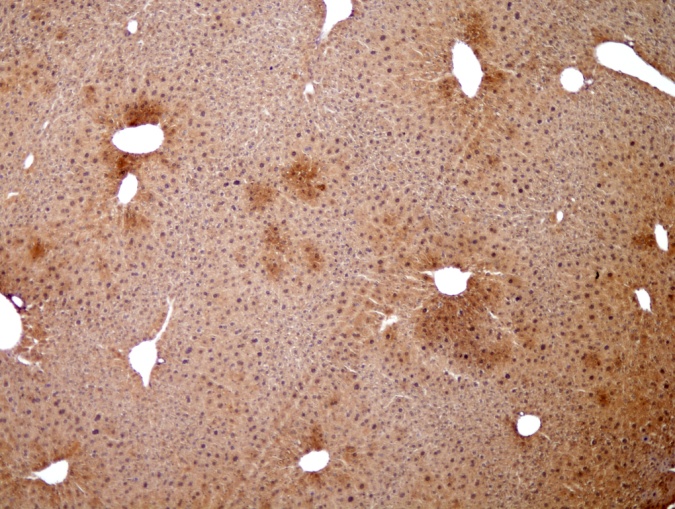

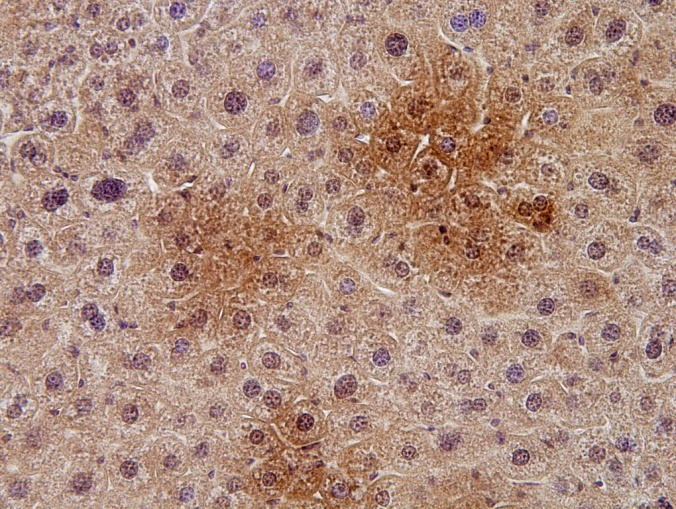


**p75NTR**

**Figure S6.** Immunohistochemistry showing parenchymal localization of TrkA and p75NTR peptides in normal mouse livers. Formalin-fixed and paraffin-embedded mouse liver tissues were subjected to immunohistochemical staining and counterstaining with hematoxylin. Note that homogenous pattern and sparsely spotted distribution of TrkA and p75 NTR, two NGF cognate receptors, were seen in parenchyma of normal mouse livers. Images at right panel are the magnified rectangular area indicated by dashed lines in left images. Bars = 100 μm.
